# Functional impacts of lactylation in Hypoxia‒primed mesenchymal stromal cells

**DOI:** 10.3389/fcell.2025.1678282

**Published:** 2025-10-24

**Authors:** Fanyi Zhao, Qixing Tang, Jie Liu

**Affiliations:** ^1^ Medicine School, Kunming University of Science and Technology, Kunming, Yunnan, China; ^2^ Regenerative Medicine Research Center, The First People’s Hospital of Yunnan Province, Kunming, Yunnan, China; ^3^ The Affiliated Hospital of Kunming University of Science and Technology, Kunming, Yunnan, China

**Keywords:** mesenchymal stromal cells, hypoxic microenvironment, lactylation, regenerative medicine, metabolic reprogramming

## Abstract

Hypoxic culture (1–5% O_2_) significantly enhances the biological activity and therapeutic potential of mesenchymal stromal cells (MSCs) by activating the HIF-1α signaling pathway. This activation promotes stemness maintenance, enhances proliferative capacity, and improves immunomodulatory functions, such as upregulating the secretion of indoleamine 2,3‒dioxygenase (IDO) and prostaglandin E2 (PGE2). Furthermore, hypoxia optimizes paracrine effects through modulating the release of vascular endothelial growth factor (VEGF) and hepatocyte growth factor (HGF), while also improving cell homing and post-transplantation survival rates. Under hypoxic conditions, MSCs primarily rely on glycolytic metabolism, resulting in lactate accumulation. This lactate serves not only as a metabolic byproduct but also as a precursor for lactylation, a novel form of epigenetic modification. Given the limited research on MSC-specific metabolic mechanisms driven by lactylation, investigating lactylation modifications‒such as histone H3 lysine 18 lactylation (H3K18la)‒and their impact on MSCs function is crucial. We propose that the ‘hypoxia-lactate-lactylation’ axis represents a key metabolic-epigenetic mechanism that may further enhance immunomodulatory and tissue‒repair capabilities via epigenetic regulation, offering novel targets for metabolic intervention in clinical cell therapy. This approach could maximize the therapeutic potential of MSCs in clinical applications, with a high safety profile that avoids risks such as tumorigenicity, donor-dependent variability, and senescence.

## 1 Introduction

Mesenchymal stromal cells (MSCs) hold significant therapeutic value in immunomodulatory functions and cell-based therapies due to their remarkable paracrine capacity, multipotent differentiation potential, and immunomodulatory properties. However, conventional *in vitro* expansion under ambient oxygen tension (21% O_2_) often leads to loss of stemness and functional impairment, limiting clinical efficacy. Studies demonstrate that hypoxic culture (1–5% O_2_) recapitulates the physiological microenvironment by activating hypoxia‒inducible factor‒1α (HIF‒1α). This activation enhances key MSCs properties, including stemness maintenance, proliferative capacity, differentiation potential, migratory ability, and paracrine activity, while simultaneously optimizing their immunomodulatory functions (Sun et al., 2020; Kanichai et al., 2008; Xu et al., 2019; Lan et al., 2015; Noronha et al., 2019; Zhu et al., 2023). While the tumor-promoting roles of MSCs had also been reported previously (Melze et al., 2016). However, hypoxia triggers metabolic reprogramming in MSCs, shifting from oxidative phosphorylation to glycolysis and results in substantial lactate accumulation. Established evidence indicates that lactate serves as a key mediator in the immunomodulatory effects of human umbilical cord MSCs (huc-MSCs) (Selleri et al., 2016). A novel immunosuppressive pathway in MSCs was first described in 2023, which functions independently from the classical immunomodulatory mechanism that relies on glycolysis-derived lactate metabolites (Pradenas et al., 2023). This phenomenon not only impacts the cellular microenvironment but may also regulate MSCs functionality through a novel post‒translational modification: lactylation.

Lactylation is a recently discovered epigenetic regulatory mechanism wherein lactate acts as a substrate to covalently modify histones (e.g., H3K18la) or non‒histone proteins, thereby modulating gene expression (Zhang et al., 2019). In tumor and immune cells, lactylation regulates inflammatory responses, metabolic adaptation, and cell fate determination. However, research on lactylation’s modulation of MSCs biological functions under hypoxic remains nascent. Current evidence suggests that lactylation may enhance MSCs therapeutic potential by upregulating immunomodulatory molecules, promoting tissue‒repair factor secretion, and improving homing and engraftment efficiency (Xie et al., 2023). Furthermore, aberrant lactylation accumulation may induce metabolic stress and compromise MSCs safety. Precise regulation of lactylation levels is therefore critical for optimizing cell‒based therapeutic strategies. This review aims to explore the emerging role of the ‘hypoxia-lactate-lactylation’ axis in modulating MSCs biology.

### 1.1 Background introduction

#### 1.1.1 Definition, origin, and primary functions of MSCs

In 2025, Yan et al. revealed the difference for the first time between MSCs and stem cells through single‒cell transcriptomic analysis (Yan et al., 2025). This study redefined biomarkers to discriminate these populations and laid the foundation for updating MSCs standards. Subsequently, the Delphi study was used to reformulate the definition of MSCs, resulting in the retention of nine items as core criteria after multiple deliberation rounds (Renesme et al., 2025). At its 2025 annual meeting, the International Society for Cellular Therapy (ISCT) published revised MSC identification criteria, explicitly defining MSCs as *mesenchymal stromal cells* (Renesme et al., 2025). The defining markers now must include positive markers (CD73^+^, CD90^+^, CD105+) and negative markers (CD45‒), while eliminating the 2006 criteria for demonstrating trilineage differentiation potential and adherent growth under standard culture conditions (Renesme et al., 2025; Dominici et al., 2006). The ability to differentiate is a key part of their functional identity, even if it's not used solely for definition anymore. Furthermore, the updated standards emphasizes the need to indicate the source of the organization. Critically, use of the term “stem” (i.e., *mesenchymal stem cells*) requires experimental evidence demonstrating stemness (Renesme et al., 2025).

MSCs can be isolated from diverse tissue sources, including bone marrow, adipose tissue, umbilical cord blood, fetal blood, placenta, dental pulp, Wharton’s jelly, skeletal muscle, dermis, and menstrual blood‒derived endometrial tissue, etc (Thirumala et al., 2009; Zuk et al., 2001; Erices et al., 2000; Campagnoli et al., 2001; In 't Anker et al., 2004; Gronthos et al., 2000; Wang et al., 2004; Kita et al., 2010; Fernández-Santos et al., 2022). The Delphi study further identifies dental follicle as an MSCs source (Renesme et al., 2025). These cells exhibit robust and critical functions in tissue homeostasis, injury repair, and immunomodulation (Miao et al., 2006). Their therapeutic efficacy in tissue injury repair, homeostasis maintenance, anti‒inflammatory responses, immunomodulation, and regenerative medicine stem from their multi-potent differentiation capacity and paracrine activity. Recent studies reveal their trans‒lineage differentiation potential: under specific *in vitro* culture conditions, MSCs undergo ectodermal differentiation (e.g., neural lineage cells expressing βIII‒tubulin and microtubule-Associated Protein 2 [MAP2]) and endodermal differentiation (e.g., hepatocyte‒like cells exhibiting albumin secretion and cytochrome P450 family three subfamily a polypeptide 4 [CYP3A4] activity) (Ababneh et al., 2022). Capitalizing on these distinctive properties, MSCs have emerged as a leading cellular therapeutic strategy in clinical applications (Alwohoush et al., 2024).

#### 1.1.2 Functional properties and mechanisms of MSCs

The functional properties of MSCs are attributed to their paracrine effect, immunomodulatory properties, and tissue regenerative functions. These cells exert their therapeutic effects through multiple mechanisms, including: paracrine signaling, cell‒cell interactions, mitochondrial transfer and epigenetic regulation.

MSCs’ classical capacity for trilineage differentiation–encompassing ostegenic, chondrogenic, and adipogenic lineages (mesodermal derivatives)–is pivotal for tissue regeneration. Experimental evidence demonstrates that directed *in vitro* differentiation for 1–3 weeks induced MSCs commitment to chondrocytes, osteoblasts, and adipocytes. Notably, extended culture (three to four weeks) reveals trans‒lineage plasticity through differentiation into functional cardiomyocyte‒like cells, which express troponin T and contract spontaneously (Zhidu et al., 2024; Pittenger et al., 1999). A seminal 2008 study identified key signaling pathways governing MSCs differentiation, identifying activin‒mediated transforming growth factor–β (TGF–β) signaling, platelet‒derived growth factor (PDGF) receptor cascades, and fibroblast growth factor (FGF) mitogenic pathways as critical regulators of lineage commitment (Ng et al., 2008).

In addition, extracellular vesicles and exosomes are now recognized as key mediators of the regenerative and immunomodulatory functions of MSCs. MSCs mediate repair and regeneration of damaged cells and tissues through two principal mechanisms (Sun et al., 2020): lineage‒specific differentiation into tissue‒resident cell types to replace damaged cells (Kanichai et al., 2008). Membrane fusion‒mediated cytoprotection via direct cell‒cell contact, facilitating organelle/cytoplasmic transfer to rescue compromised or apoptotic cells (Tao et al., 2016; Watt et al., 2013; Spees et al., 2016). Furthermore, MSCs paracrine activity orchestrates regeneration through bioactive molecules that promote: (i) angiogenesis (e.g., VEGF, angiopoietin-1 [ANG‒1]), (ii) extracellular matrix remodeling (e.g., HGF, insulin-like growth factor-1 [IGF‒1]), (iii) anti‒fibrotic responses (e.g., tumor necrosis factor-inducible gene six protein [TSG‒6]), and (iv) immunoregulation (e.g., stromal cell-derived factor-1/C-X-C motif chemokine ligand 12 [SDF‒1/CXCL12]) (Ong and Dilley, 2018; Mihaylova et al., 2018; Hamid et al., 2022; Zacharek et al., 2007; Keshavarz et al., 2019; Meng et al., 2021; Katagiri et al., 2017). Beyond direct differentiation, MSCs facilitate repair via: (i) intercellular organelle transfer (notably mitochondria) and (ii) tunneling nanotube (TNT)‒mediated molecular trafficking (Figeac et al., 2014; Feng et al., 2019). Additionally, their intrinsic homing capacity enables targeted migration to injury through chemokine receptor‒dependent sensing of inflammatory mediators (Song and Li, 2011; Imitola et al., 2004).

Under pathological conditions, MSCs regulate immunity through dual mechanisms: (Sun et al., 2020): paracrine signaling mediated by extracellular vesicles and soluble factors, which suppresses pro‒inflammatory T cells/natural killer (NK) cells while promoting regulatory T cells (Tregs) expansion; (Kanichai et al., 2008); direct cell‒contact interactions that modulate B cell maturation and drive macrophage polarization toward anti‒inflammatory (alternatively activated macrophage [M2]) phenotypes (Li et al., 2021). Mechanistically, MSC-derived extracellular vesicles, particularly exosomes, together with secreted immunomodulatory factors, collectively establish an immunosuppressive niche. These vesicles transfer a variety of bioactive molecules, including: interleukin-10 (IL‒10) (anti‒inflammatory cytokine), interleukin-11 (IL‒11) (tissue‒protective signaling), PGE2 (myeloid suppression), TGF‒β (Treg induction), programmed cell death ligand 1/2 (PD‒L1/2) (T cell exhaustion induction), and IDO (tryptophan catabolism‒mediated suppression). These collectively dampen excessive immune activation (Li et al., 2021; Cho et al., 2024; Kulesza et al., 2023; Huang et al., 2024; Putra et al., 2018; Davies et al., 2017; Su et al., 2014).

The therapeutic value of MSCs stems from their dual capabilities: robust proliferative capacity *in vitro* and multipotent differentiation into clinically relevant cell lineages, enabling tissue maintenance and regeneration. Furthermore, their potent immunomodulatory properties make them as promising therapeutic agents for treating various diseases.

In hepatic disorders, huc‒MSCs exert therapeutic effect by suppressing hepatic stellate cell (HSC) activation, delivering cytoprotective factors via exosome, and counteracting oxidative stress‒induced hepatocyte injury (Shi et al., 2024; Xie et al., 2020). For systemic lupus erythematosus (SLE), huc-MSCs immunotherapy demonstrate clinical efficacy through immune tolerance restoration, reduced auto-antibody production, and attenuated end‒organ damage, with reported survival rates exceeding 80% (Wang et al., 2018). In the treatment of inflammatory arthritis, huc‒MSCs mediate disease‒modifying effects via osteochondral differentiation, micro-environment reprogramming through anti‒inflammatory cytokines secreting. (Ma et al., 2019; Gu et al., 2015; Dao et al., 2019). For the treatment of cerebrovascular diseases (stroke, traumatic brain injury), huc‒MSCs mitigate injury through VEGF‒mediated revascularization, trophic factor induction (glial cell line-derived neurotrophic factor [GDNF], brain-derived neurotrophic factor [BDNF]) reducing neuronal apoptosis, synaptic plasticity support, pro‒inflammatory cytokines suppression to restore neural circuitry and motor function (Peng et al., 2015; Wang et al., 2013; Qi et al., 2018). In cardiovascular diseases, huc‒MSCs drive cardiac repair by promoting cardiomyocyte regeneration, enhancing neovascularization, modulating cytokine storm (Lim et al., 2018).

Variability in MSCs clinical efficacy is attributed to donor‒related factors, including tissue source and intrinsic biological differences. Proteomic analysis of equine MSCs secretomes (314 identified proteins) revealed that donor age and tissue origin significantly influence protein composition, potentially impacting therapeutic outcomes (Turlo et al., 2023). Bone marrow‒derived MSCs (BMSCs) and adipose‒derived MSCs (ADSCs) are extensively utilized in cell‒based therapies due to their compatibility with both autologous and allogeneic applications (Liu Y. et al., 2024). Key clinical advantages include accessibility, therapeutic versatility, and regulatory progress evidenced by > 500 registered clinical trials. BMSCs were the first MSCs population used clinically. However, donor age significantly reduces BMSC yield, cellular quality, and multipotent differentiation capacity (Kern et al., 2006; Bagge et al., 2022). Furthermore, the highly invasive nature of bone marrow aspiration often causes significant patient morbidity (Thirumala et al., 2009). Suboptimal therapeutic efficacy has contributed to BMSC failures in several Phase III clinical trials (Liao et al., 2017). While tissue origin drives MSCs heterogeneity, donor‒specific factors (age, metabolic status) and *ex vivo* manipulations (prolonged culture‒induced senescence, oxygen tension shifts) further amplify their therapeutic potential diversity (Turlo et al., 2023; Liu Y. et al., 2024). Thus, fine‒tuning MSCs phenotypes through preconditioning strategies, epigenetic modulation, or biomechanical priming represents a promising approach to overcome current limitations in cell‒based therapies.

### 1.2 Reasearch meaning

Hypoxic preconditioning (1–5% O_2_) recapitulates physiological O_2_ tension in native stem cell niches (e.g., bone marrow, umbilical cord), inducing HIF‒1α‒mediated transcriptional reprogramming that enhances MSCs therapeutic efficacy. Mechanistically, hypoxia stabilizes HIF‒1α by inhibiting prolyl hydroxylase (PHD)‒dependent degradation. This stabilization upregulates pluripotency markers (Oct4, Nanog), suppresses differentiation‒related genes, maintains MSCs in an undifferentiated state. HIF‒1α further enhances proliferative capacity through dual regulation of metabolic reprogramming and cell cycle progression. Regarding immunomodulation, HIF‒1α potentiates MSC‒mediated immunosuppression by activating immune checkpoints and enhancing paracrine activity. Specifically, HIF‒1α upregulates IDO and PGE2 via the cyclooxygenase-2/prostaglandin E2 (COX‒2/PGE2) pathway, depleting local tryptophan while increasing immunosuppressive kynurenines. This inhibits T‒cell proliferation and polarizes macrophages toward regulatory phenotypes. Besides, HIF‒1α drives the secretion of VEGF and HGF to facilitate tissue repair. Hypoxic preconditioning also upregulates homing receptors (e.g., C-X-C motif chemokine receptor 4 [CXCR4]), and modulates apoptotic pathways to enhance the therapeutic efficacy of MSCs. Specifically, Hypoxia induces CXCR4 expression throutgh HIF‒1α binding to the CXCR4 promoter, potentiating SDF‒1/CXCR4‒mediated chemotaxis. Anti‒apoptotic programming occurs via upregulating the expression of B‒cell lymphoma‒2 (Bcl‒2) and downregulating the expression of pro‒apoptotic Bcl‒2 Associated X‒protein (Bax) (Feng and Wang, 2017). This dual regulation significantly improves post‒transplantation cell survival. However, severe hypoxia can impair the *in vitro* therapeutic efficacy of MSCs and promote their senescence and apoptosis (Jaraba-Álvarez et al., 2025; Khasawneh and Abu-El-Rub, 2022).

## 2 Hypoxic culture: principles and impacts

### 2.1 Description of hypoxic culture conditions

Hypoxia is defined as a pathophysiological state and characterized by inadequate oxygen supply to tissues/cells or excessive oxygen consumption, resulting in subphysiological oxygen tension (typically <5% O_2_ in most tissues) (MacIntyre, 2014; Wang X. et al., 2022). Hypoxia can be classified into three principal categories: systemic hypoxia, localized hypoxia, and functional hypoxia. The underlying mechanisms involve (Sun et al., 2020): hypoxemia (reduced arterial oxygen saturation); (Kanichai et al., 2008); impaired tissue oxygen delivery (due to circulatory insufficiency or hemoglobin dysfunction) (Xu et al., 2019); defective cellular oxygen utilization (Hypoxemia vs. hypoxia, 1966; Mallat et al., 2022; Østergaard et al., 2015). In stem cell biology, oxygen concentration critically regulates biological properties through hypoxia‒inducible factor (HIF)‒mediated pathways, influencing pluripotency maintenance, differentiation potential, and metabolic reprogramming (glycolytic shift) (Xin et al., 2023).

### 2.2 Hypoxic conditioning of MSCs: Mechanisms and therapeutic implications

The hypoxic environment shifts the metabolic process of MSCs toward glycolysis, leading to excessive lactate production. It is well established that lactate derived from MSCs serves as a key mediator in regulating immune function. Furthermore, hypoxic preconditioning modulates MSCs proliferation, differentiation, migration, and angiogenesis while also enhancing homing potential, suppressing apoptosis and inflammation, improving post‒transplantation survival, increasing stress tolerance, and augmenting therapeutic efficacy (Pouikli et al., 2022; Rosová et al., 2008; Gupta et al., 2022; Yang Y. et al., 2022; Tang et al., 2005). Furthermore, hypoxia reduces reactive oxygen species (ROS) generation, mitigating cellular damage and necrosis in MSCs (Bertram and Hass, 2008). Mechanistically, whether lactate acts as a primary mediator influencing these functional alterations remains debatable. What is certain, however, is that hypoxia (1–5% O_2_) upregulates key angiogenesis and vasoactivity regulators including: angiopoietin (ANG), VEGF, basic fibroblast growth factor (bFGF), transforming growth factor‒β1 (TGF‒β1), monocyte chemoattractant protein‒1 (MCP‒1), tissue inhibitor of metalloproteinase‒1 (TIMP‒1), matrix metallopeptidase‒9 (MMP‒9), and chemokine ligand 20 (CCL20) (Chen et al., 2014; Xia et al., 2018; Quade et al., 2020). Hypoxic preconditioning further elevates the expression of VEGF, TGF‒β1, IGF‒1, fibroblast growth factor 10 (FGF10), and epidermal growth factor (EGF). These factors synergize with exsome-contained miRNA to activate (Sun et al., 2020): the phosphatidylinositol 3-kinase/protein kinase B (PI3K/AKT) pathway to promote cell survival, proliferation, and migration (Kanichai et al., 2008); the transforming growth factor-beta/mothers against decapentaplegic homolog 2 (TGF‒β/SMAD2) pathway to regulate anti‒apoptotic and pro‒regenerative responses (Vizoso et al., 2017; Jung et al., 2007; Chang et al., 2013). Critically, hypoxic’s modulation of MSCs function is a double‒edged sword, with effects determined by oxygen concentration (hypoxia severity) and exposure duration.

Extensive studies document hypoxia‒induced alterations in MSCs biological functions across varying oxygen tensions.Hypoxia‒inducible miR‒486 enhances PI3K/AKT signaling activity via targeted phosphatase and tensin homolog (PTEN) suppression, promoting BMSC proliferation and survival (Shi et al., 2016). Normoxia (21% O_2_) irreversibly impairs MSCs functionality and osteogenic differentiation capacity, promoting exploration of hypoxic conditioning (Pouikli et al., 2022). Acute hypoxia (1% O_2_) significantly enhances BMSC migration and angiogenesis, while hypoxic preconditioning robustly stimulates proliferation and multilineage differentiation (Annabi et al., 2003; Ren et al., 2006; Xie et al., 2006). Moderate hypoxia (5% O_2_) elicts a biphasic proliferative response: reduced cell numbers in primary cultures but enhanced expansion in passaged cells (Ejtehadifar et al., 2015). hMSCs under moderate hypoxia (2% O_2_) exhibit prolonged lag phases but sustained proliferation, with elevated colony‒forming unit (CFU) capacity and stemness‒related gene expression (Grayson et al., 2006). Mechanistically, hypoxia induces MSCs proliferation via activation of the PI3K/AKT signaling (evidenced by elevated p‒AKT levels) (Sheng et al., 2017), further potentiated by: angiotensin II type 1 (AT1) receptor‒mediated PI3K activation in murine MSCs under hypoxia (3% O_2_) (Zhang et al., 2015) and SNHG16 lncRNA modulation in human placenta‒derived MSCs (hP‒MSCs) (Feng et al., 2022). However, contrasting findings were reported that severe hypoxia (1% O_2_) transiently reduces induced MSCs (iMSCs) proliferation/viability, yet prolonged exposure (50 h) yields superior iMSC growth compared to normoxic cultures (Alwohoush et al., 2024). These collective results suggest that the proliferative response of MSCs to hypoxia is multifactorial, governed by oxygen concentration gradient, exposure duration and cell type‒specific adaptations.

The multipotent differentiation potential of MSCs is significantly modulated by hypoxic conditions. Substantial evidence indicates that hypoxia consistently promotes chondrogenic differentiation in MSCs (Yang Y. et al., 2022). In contrast, its effects on adipogenic and osteogenic differentiation demonstrate context‒dependent regulation, with either stimulatory or inhibitory outcomes depending on specific experimental conditions. Mechanistically, hypoxic treatment of BMSCs enhances citrate carrier (CiC) activity, which prevents acetyl‒Coenzyme A (CoA) accumulation in mitochondria and subsequently promotes histone acetylation. Additionally, hypoxia reduces chromatin condensation at osteogenic gene promoter and enhancer. These coordinated epigenetic modifications collectively enhance osteogenic differentiation capacity under hypoxic conditions (Pouikli et al., 2022).

Hypoxic conditioning significantly enhances the paracrine activity of MSCs. Moreover, under hypoxic conditions, MSCs extensively use exosomes for intercellular communication. These vesicles deliver miRNAs and proteins to modulate the relevant cytokines expression (Yuan et al., 2025; Pulido-Escribano et al., 2022), thereby orchestrates long-distance cellular responses that promote angiogenesis, cell survival, and tissue regeneration. The hypoxia‒induced MSCs secretome plays crucial roles in promoting angiogenesis, suppressing inflammatory responses, and providing cytoprotective effects against apoptosis. Regarding angiogenic mechanisms, MSCs contribute to neovascularization through two principal pathways: (Sun et al., 2020): direct differentiation into vascular smooth muscle cells (SMCs) and endothelial cells (ECs), (Kanichai et al., 2008), paracrine regulation via intercellular communication with ECs and secretion of pro‒angiogenic factors (Hegde et al., 2024). At the molecular level, hypoxia triggers HIF‒1α stabilization and nuclear accumulation in endothelial cells. Activated HIF‒1α binds to VEGF promoters, up-regulating its transcription and subsequent pro‒angiogenic activity (Ahluwalia and Tarnawski, 2012). 24‒hour hypoxic preconditioning (1.5% O_2_) significantly increases the expression of erythropoietin receptor (EPOR) and VEGF in mouse-derived MSCs (mdMSCs) compared to versus controls (Lan et al., 2015). Current research demonstrate that hypoxic preconditioning activates HIF‒α signaling, coordinates the expression of VEGF and its cognate receptors vascular endothelial growth factor receptor 1/2 (VEGFR1/2), EPOR, and ANG-1 (Hu et al., 2008). This HIF‒mediated transcriptional program represents the fundamental mechanism underlyig hypoxia‒induced angiogenic potentiation in MSCs.

Li et al. (2023) demonstrated that 24‒hour hypoxic preconditioning (2% O_2_) significantly enhances the immunosuppressive properties of huc-MSCs, particularly their anti‒inflammatory capacity (Li et al., 2023). Regarding cytoprotective mechanisms, Li et al. reported that hypoxia‒treated MSCs (1% O_2_) elevated the expression of pro‒survival factors (AKT kinase and p‒AKT, hypoxia-inducible factor-alpha [HIF‒α]) and activated key cytoprotective mediators in target cells (anti‒apoptotic proteinsBcl‒2, Caspase‒3 inhibitors, metallothionein-2 [MTP‒2], TGF‒β1) (Li et al., 2017). As noted, lactate produced by MSCs has been reported to regulate the immunosuppressive functions via a novel alternative pathway (Pradenas et al., 2023). Furthermore, hypoxia‒preconditioned MSCs exhibit enhanced antioxidant capacity. These findings collectively establish hypoxic preconditioning as an effective strategy to enhance MSC‒mediated cytoprotection against various stressors.

## 3 Concept and biological significance of lactylation modification

### 3.1 Definition of lactylation modification

Post‒translational modifications (PTMs) critically regulate protein conformation, activity, and function, participating nearly all cellular pathways. These modifications drive diverse physiological and pathological processes while maintaining cellular homeostasis. Histones‒core structural components of nucleosomes‒consist of five major classes (H1, H2A, H2B, H3, and H4). Characterized by structured globular domains and flexible N‒terminal tails, histones are particularly susceptible to modifications at their tail regions, with the N‒terminus serving as the primary modification site. Enzymatic PTMs of histones are essential regulators of gene expression, chromatin architecture, and cellular functions. Common histone PTMs include methylation, acetylation, phosphorylation, ubiquitination, lactylation, and carboxylation.

Lactylation is a recently discovered, functionally significant PTMs. In 2019, Yingming Zhao’s research group (University of Chicago) first identified lysine lactylation (Kla) as a novel histone mark induced by lactate (Zhang et al., 2019). Their seminal work mapped 28 lactylation sites on core histones in both human and murine cells. Histone lactylation predominantly involves L‒lactate covalently modifying lysine residues through lactyl group addition, thereby regulating gene transcription (Wang N. et al., 2022; Zhan et al., 2021). Emerging evidence indicates histone acetyltransferase p300 participates in H3 lactylation modification (Hu et al., 2024). Both knockdown and overexpression experiments in mouse bone marrow‒derived macrophages and germinal vesicle (GV) oocytes demonstrate P300’s regulatory role in histone lactylation dynamics (Cui et al., 2021; Lin et al., 2022). This PTMs establishes a molecular link between lactate metabolism, transcriptional regulation, and epigenetics (Yu et al., 2024). Current research reveals that protein lactylation plays crucial roles in metabolic regulation, cell cycle control, protein function and stability, signal transduction, cellular stress responses, and tumor microenvironment (TME) modulation (Liu et al., 2023; Niu et al., 2021; Izzo and Wellen, 2019). Furthermore, lactylation modifications are implicated in various pathological conditions, including malignancies, inflammatory disorders, psychiatric diseases, infectious diseases, neurodegenerative conditions, and metabolic dysregulation (Sun et al., 2023; Yao et al., 2024; Li et al., 2022). Therefore, investigating lactylation deepens our understanding of fundermental cellular regulatory mechanisms. Under hypoxic or hyperglycemic, cells adapt to hypoxia through glycolytic reprogramming, increasing lactate production. Subsequent histone lactation then links metabolic states to gene regulation.

**FIGURE 1:**
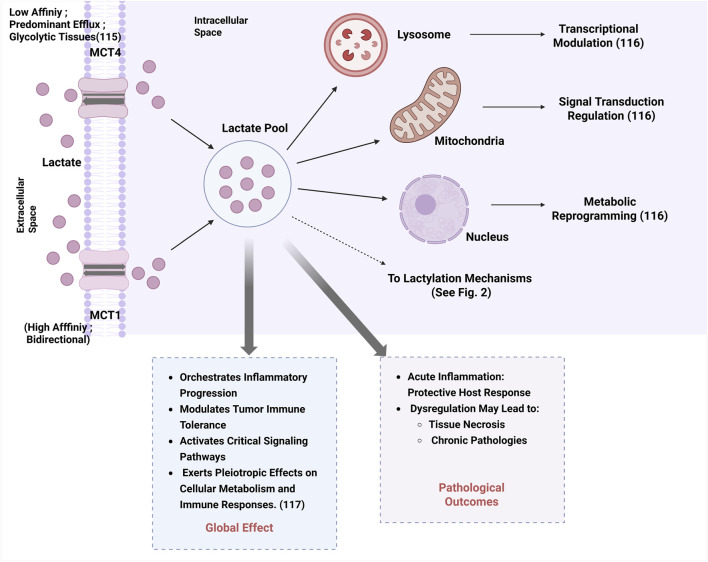
Lactate transport, intracellular accumulation, and global cellular effects.

Lactate, a key metabolic intermediate, functions both as a post‒translational modification mediator and a metabolic regulator. In mammalian systems, lactate transport is primarily mediated by monocarboxylate transporters (MCTs), with distinct functional properties. monocarboxylate transporter 1 (MCT1) exhibits the highest affinity for lactate and functions as a bidirectional transporter dependent on substrate concentration gradients. In contrast, monocarboxylate transporter 4 (MCT4) is predominantly expressed in highly glycolytic tissues (e.g., tumors), specialized for lactate efflux despite its bidirectional transport capability (Beloueche-Babari et al., 2020). Intracellular lactate accumulation in lysosomes, mitochondria, and nuclei regulates multiple cellular processes through transcriptional modulation, signal transduction regulation, and metabolic reprogramming (Ivashkiv, 2020). Elevated lactate levels exert pleiotropic effect on cellular metabolism and immune responses via orchestrating inflammatory progression, modulating tumor immune tolerance, and activating critical signaling cascades (Zha et al., 2024), as summarized in Figure 1. While acute inflammation serves as a protective host response, its dysregulation may progress to tissue necrosis and chronic pathologies.

**FIGURE 2:**
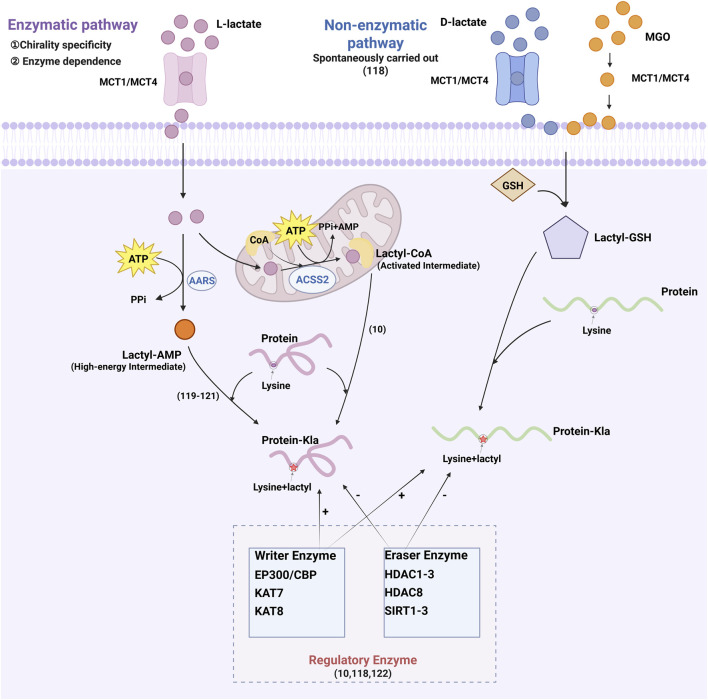
Lactic acid transport and protein lactylation modification mechanism.

As shown in Figure 2, protein lactylation are mediated by two distinct mechanisms: enzymatic and non‒enzymatic pathways. While both utilize lactate as a common substrate, they differ in chiral specificity and biochemical requirements (He et al., 2024). Enzymatic lactylation primarily utilizes L‒lactate and occurs via two distinct pathways. The first pathway converts L‒lactate into L‒lactyl‒CoA, which serves as the direct substrate for lactylation. This activated intermediate facilitates lactyl group transfer to lysine residues on target proteins (Zhang et al., 2019). The second pathway is mediated by aminoacyl‒tRNA synthetases (alanyl-tRNA synthetase 1 [AARS1] and alanyl-tRNA synthetase 2 [AARS2]), which directly couple L‒lactate with adenosine triphosphate (ATP) to generate lactyl‒adenosine monophosphate (AMP). This high‒energy intermediate subsequently donates the lactyl group to lysine residues, yielding lactylation (Mao et al., 2024; Ju et al., 2024; Zong et al., 2024). In contrast, non‒enzymatic lactylation is mediated by D‒lactate, a intermediate of glycolysis. This process utilizes methylglyoxal (MGO) that reacts with glutathione to produce lactylglutathione, the direct substrate for non‒enzymatic lactylation. Unlike enzymatic lactylation, this mechanism operates independently of specific transferases, depending instead on spontaneous chemical modifications. Several enzymes regulate histone lactylation, including EP300 and its homolog CREB‒binding protein (CBP), lysine acetyltransferases (lysine acetyltransferase 7 [KAT7] and lysine acetyltransferase 8 [KAT8]), histone deacetylases (histone deacetylase 1, 2, 3 [HDAC1–3] and histone deacetylase 8 [HDAC8]), and sirtuins (sirtuin 1, 2, 3 [SIRT1–3]) (Zhang et al., 2019; He et al., 2024; Yang K. et al., 2022), which collectively link metabolic flux to epigenetic regulation through lactylation control.

### 3.2 Lactylation mechanisms in physiological contexts

**FIGURE 3:**
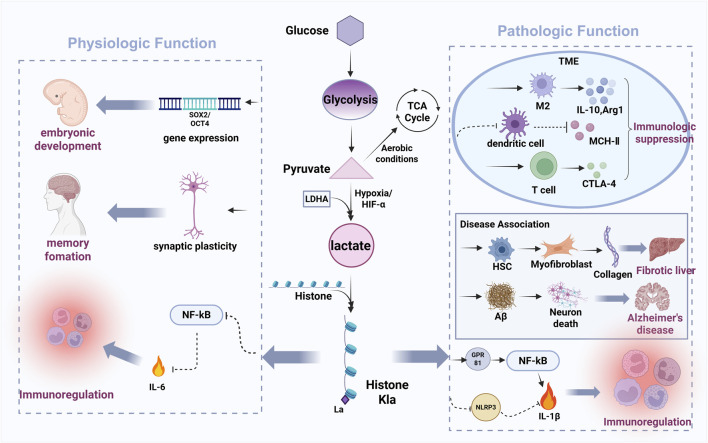
The bidirectional regulatory role of lactylation in physiological and pathological processes.

As described in Figure 3, emerging evidence establishes lactylation as a key regulator of physiological processes including embryonic development, cell division, cellular differentiation, angiogenesis, and memory formation under normal physiological conditions (Li et al., 2022; Wang J. et al., 2024; Yang et al., 2021). Yang et al. (2021) first characterized the dynamic pattens of histone lactylation (histone H3 lysine 23 lactylation [H3K23la], H3K18la, and pan‒histone lactylation) during mouse oocyte maturation and preimplantation embryonic development (Yang et al., 2021). Their works revealed that these modifications were enriched in germinal vesicle (GV)‒stage oocytes but declined post‒fertilization, with oxygen tension identified as a critical modulator (Yang et al., 2021). In embryonic stem cells (ESCs), lactate supplementation upregulates germline and zygotic genome activation (ZGA)‒related genes (particularly Zscan4) through H3K18la accumulation at these loci, where lactylated co-factors promote transcriptional elongation (Tian and Zhou, 2022; Xie et al., 2022). Conversely, Lin et al. demonstrated that in mouse oocytes, Tfap2α over-expression elevates p300 expression, increasing global histone lactylation levels‒such as H3K18la, histone H4 lysine 12 lactylation (H4K12la) and pan‒Kla‒and impairing spindle assembly and chromosomal alignment (Lin et al., 2022). Beyond histones, lactylation regulates non‒histone proteins like Yin Yang‒1 (YY1). Wang et al. reported hypoxia‒induced YY1‒lysine 183 lactylation (K183la) activates fibroblast growth factor 2 (FGF2) transcription to drive retinal neovascularization (Wang X. et al., 2023). Additionally, Descalzi’s et al. (2019) revealed that astrocyte‒derived lactate mediates memory consolidation by enhancing neuronal mRNA translation and Arc/Arg3.1 expression (Descalzi et al., 2019). It should be noted that the molecular mechanisms by which hypoxia-induced histone lactylation influences MSCs function remain insufficiently explored. A study by Chen et al. reported that chronic intermittent hypoxia (CIH) enhances glycolysis and lactate production in mouse BMSCs, leading to increased H3K18la levels at the proliferator‒activated receptor gamma (PPARγ) promoter region. This epigenetic modification promotes PPARγ transcription and subsequently impairs osteogenic differentiation (Chen et al., 2025). In contrast, another study demonstrated that a 3D-printed polycaprolactone/nano-hydroxyapatite (PCL/nHA) scaffold enabling sustained lactate release (mimicking hypoxia conditions) promotes signal transducer and transcription 1, lysine 193 (STAT1-K193) lactylation, which in turn releases runt-related transcription factor 2 (Runx2) and enhances osteogenic transcription in human BMSCs (Zeng et al., 2025).

### 3.3 Lactylation mechanisms in cancer cells and the TME

Figure 3 has shown that the Warburg effect, a hallmark of cancer metabolism, describes tumor cells’ preferential use of glycolysis over oxidative phosphorylation for energy production, even under oxygen‒rich conditions (Hanahan and Weinberg, 2011; Koppenol et al., 2011; Yu et al., 2021). This metabolic reprogramming results in substantial lactate accumulation, which functions as both a key metabolic intermediate and a signaling molecule within the TME. Throuth its regulation of gene transcription and protein function, lactylation drives metabolic reprogramming that enables tumor adaption to nutrient deprivation and sustains proliferative capacity (He et al., 2024). This suggests histone lactylation is frequently dysregulated in cancer, representing a promising therapeutic target. Within the TME, abundant lactate serves as the substrate for lactylation modifications in both tumor and infiltrating immune cells. Lactate is known to modulate immune cell behavior, including cytotoxic T-lymphocyte-associated protein 4 (CTLA‒4) upregulation in T cells, macrophage polarization, and dendritic cell immunosuppression. Pharmacologically, sodium dichloroacetate (DCA) and oxamate suppress lactate production by inhibiting the activity of pyruvate dehydrogenase (PDH) and lactate dehydrogenase (LDH), thereby reducing intracellular lactate and Kla (lysine lactylation) modifications (Zhang et al., 2019). In contrast, rotenone enhances glycolysis by blocking mitochondrial respiration, increasing both lactate and Kla levels (Zhang et al., 2019). Zhang et al. first elucidated the impact of histone lactylation on macrophage polarization, showing lactate dehydrogenase A (LDHA)‒knockout reduces lactate production, histone Kla levels, and M2 marker Arg1, while lactate supplementation increases Arg1 and Vegfa (both M2‒like macrophage associated genes) (Zhang et al., 2019). These findings indicate histone lactylation promotes Arg‒1 and wound‒healing gene expression, facilitating the pro‒inflammatory classically activated macrophage (M1) to immunosuppressive M2 macrophage phenotypic switch. In malignancy, lactate and the TME critically promote tumorigenisis throuth angiogenesis, invasion, metastasis, and immune evasion (Chen et al., 2022). Importantly, histone Kla contributes to immune suppression by reinforcing M2‒like macrophage polarization, thereby inhibiting anti‒tumor immune responses. Together, these findings highlight the lactate‒lactylation axis as a critical regulator of TME immunosuppression and a viable target for anti‒cancer therapies (Chen et al., 2022; Ngwa et al., 2019).

Current research on lactylation modification primarily focuses on TME and immune cells. These findings provide a critical conceptual framework and mechanistic insights for understanding the potential role of lactylation in MSCs biology. However, it is crucial to emphasize the differences arising from cell type and metabolic status in this process.

### 3.4 Impact of protein lactylation on disease pathogenesis

Recent studies have delineated how Kla contributes to disease pathogenesis, demonstrating its capacity to either directly alter cellular signaling pathways or indirectly regulate downstream effects through upstream signaling cascades. These findings provide promising novel therapeutic targets and offer innovative approaches for modulating disease‒relevant pathways.

A 2023 study using liver biopsies from cirrhosis patients revealed that huc‒MSCs therapy significantly alters protein lactylation profiles, particularly affecting glucose metabolic enzymes, suggesting glucometabolic pathways may mediate huc‒MSCs’ therapeutic effects in cirrhosis (Xie et al., 2023). This finding demonstrates that the immunomodulatory and regenerative functions of MSCs are closely associated with metabolic reprogramming and lactylation modification.

### 3.5 Biological significance of lactylation

#### 3.5.1 Histone lactylation in gene expression regulation

Histone lactylation regulates gene expression through altering chromatin architecture, controlling transcription factor/cofactor recruitment, and directly modulating specific target gene expression. As chromatin’s fundamental structural units, histones coordinate genomic organization and transcriptional regulation through interactions with deoxyribonucleic acid (DNA) and non‒coding ribonucleic acid (nc RNA). At the molecular level, lactylation influences gene expression by altering histone charge state, interfering with transcription factor binding, and modulating transcriptional initiation and elongation. MSCs may utilize these mechanisms to regulate the expression of key functional genes. Additionally, growing evidence indicates lactylation may indirectly modulate gene expression by influencing other PTMs status, particularly histone acetylation, forming a multi‒layered regulatory system.

#### 3.5.2 Lactylation couples cellular metabolism with gene expression

Lactylation represents a crucial epigenetic mechanism that bridges cellular metabolic states (e.g., hypoxia, enhanced glycolysis) with transcriptional regulation, effectively coupling metabolic flux with gene expression reprogramming. Furthermore, lactylation regulates the expression of metabolic pathway genes (including glycolysis and oxidative phosphorylation components), enabling cellular adaptation to metabolic stress. Hypoxic preconditioning enhances glycolytic activity in MSCs, leading to lactate accumulation and subsequent lactylation that regulates gene expression. Conversely, lactylation may further potentiate the glycolytic pathway in MSCs, thereby forming a metabolic–epigenetic cycle that promotes their adaptation to the surrounding microenvironment. Fei Li et al. found that histone lactylation promotes glycolysis by activating the transcription and expresssion of metabolic regulators. Their work in pancreatic ductal adenocarcinoma (PDAC) also revealed that H3K18la enrichment at promoter regions enhances the transcription of TTK protein kinase (TTK) and BUB1 mitotic checkpoint serine/threonine kinase B (BUB1B), which upregulates the histone acetyltransferase p300 and subsequently enhances glycolytic upregulation (Li et al., 2024a). Within the TME, this histone lactylation‒driven metabolic reprogramming further promotes oncogenesis and cancer progression. Such epigenetic regulation enables cancer cells to maintain their proliferative capacity and survival advantage under metabolic constraints.

#### 3.5.3 Lactylation in immunomodulation

Growing evidence highlights the pivotal role of lactylation in immune regulation, particularly in controlling macrophage polarization and T‒cell function. In microglia and macrophages, histone lactylation serves as a key modulator of the M1/M2 polarization balance, thereby influencing inflammatory responses and immune homeostasis. Mechanistically, lactate‒induced histone lactylation simultaneously suppresses pro‒inflammatory M1‒associated signaling pathways while promoting the transcriptional activation of anti‒inflammatory M2 phenotype genes (Ivashkiv, 2020; Xin et al., 2022). These immunomodulatory effects are further confirmed in the TME, where lactate exposure upregulates M2 markers while downregulates M1 markers in microglia (Longhitano et al., 2023). Beyond macrophages, lactylation exerts broad immunosuppressive effects by impairing cytotoxic immune cell function, which compromises both CD8^+^ T cell and natural killer T (NKT) cell anti‒tumor activity (Hao et al., 2024). Wang et al. demonstrated that in malignant pleural effusion (MPE), H3K18la promotes forkhead box protein P3 (FOXP3) expression in peripheral blood mononuclear cells (PBMCs), simultaneously enhancing the immunosuppressive function of Tregs while inhibiting NKT cell‒mediated anti‒tumor responses (Wang ZH. et al., 2023). Similarly, in glioblastoma (GBM) stem cells, histone lactylation drives immunosuppression through two coordiated mechanisms: CD47 upregulation to attenuate phagocytic activity and signal transducer and transcription 3 (STAT3) activation to reduce microglial/macrophage infiltration and impair immune surveillance (Wang S. et al., 2024). Together, these findings position lactylation as a critical epigenetic regulator that reprograms immune responses in pathological conditions.

Growing evidence demonstrates histone lactylation as a key epigenetic regulator of inflammatory gene expression that critically modulates immune cell activation and function. In GBM, lactate‒induced histone lactylation in monocyte‒derived macrophages upregulates IL‒10 expression, leading to T‒cell suppression and impaired anti-tumor immune responses (De Leo et al., 2024). Lactylation plays a critical role in M2 polarization and Treg-induced immunosuppression, providing valuable insights into its role in mediating the immunomodulatory functions of MSCs and supporting the safety of anticancer therapies.

#### 3.5.4 Lactylation dictates cellular fate

Lactylation functions as a crucial metabolic‒epigenetic regulator that governs cell lineage specification and reprogramming pathways. The transition between cellular states, particularly direct reprogramming (transdifferentiation) that bypasses pluripotent intermediates, is precisely regulated by coordinated metabolic remodeling and chromatin plasticity (Wang et al., 2021). This sophisticated process is regulated by an integrated network of transcription factors, RNA‒binding proteins, and chromatin remodelers that interact with metabolic pathways to determine cell fate (Ryall et al., 2015). CIH impairs osteogenesis and long bone growth in mouse BMSCs by modulating histone lactylation (Chen et al., 2025). In contrast, both exercise-mediated mechanical stress and sustained lactate release via 3D-printed PCL/nHA scaffolds (mimicking hypoxia condition) enhance lactylation levels, thereby promoting osteogenic differentiation in both mouse and human BMSCs (Zeng et al., 2025; Dai et al., 2025). These findings highlight the dual and the context-specific regulatory functions of lactylation in celluar reprogramming and self-renewal ability.

To provide a clear distinction between direct evidence from MSCs studies and indirect inferences from other cell systems, Table 1 summarizes the key findings on lactylation in MSCs, including study type, MSC source, lactylation target, and observed biological effects.

**TABLE 1:** Summary of direct evidence on lactylation modification in MSCs.

References	Study type	MSCs source	Lactylation target	Biological effect
Chen et al. (2025)	Experimental study (*in vitro*)	Mouse BMSCs	H3K18la at PPARγ promoter region	Impairs osteogenic differentiation
Zeng et al. (2025)	Experimental study (*in vitro*)	Human BMSCs	STAT1-K193 lactylation	Enhances osteogenic differentiation
Xie et al. (2023)	Clinical study (patient samples)	Huc-MSCs	Protein lactylation on metabolic enzymes	Alters lactylation profiles; mediates therapeutic effects in cirrhosis
Kastner et al. (2020)	Experimental study (*in vitro*)	Human BMSCs	Lactylation-mediated signaling	Enhances proliferation capacity
Selleri et al. (2016)	Experimental study (*in vitro*)	Huc-MSCs	Lactate secretion	Enhances M2-macrophage differentiation
Wu et al. (2023)	Experimental study	Mouse BMSCs	H3K18la	Promotes osteogenic differentiation
Kolodziej et al. (2019)	Experimental study (*in vitro*)	Human ADSCs	Lactylation activates PPARγ	Drives adipocyte differentiation

## 4 Hypoxia‒Driven lactylation modulates MSC functionality

Hypoxic conditioning triggers HIF‒α‒mediated transcriptional activation of glycolytic enzymes and hypoxia‒responsive genes, thereby enhancing lactate production and lactylation modification (Li Y. et al., 2024). This process profoundly influences MSCs morphology, functional adaptability, and therapeutic potential, particularly in tissue regeneration applications.

### 4.1 Morphological adaptations

Lactate‒mediated PTMs coordinate cytoskeletal remodeling through three distinct but interconnected mechanisms: (1) Direct lactylation of cytoskeletal components (including actin filaments and microtubule‒related proteins) alters their polymerization kinetics, promoting morphological transition in hypoxia‒primed MSCs from spindle‒shaped to flattened/stellate configurations that facilitate enhanced MSCs migration capacity; (2) Histone Kla at pro‒inflammatory and differentiation‒related gene loci activates epithelial-mesenchymal transition (EMT)‒related transcriptional programs, resulting in secondary cytoskeletal reorganization; and (3) Lactate‒induced lactylation of membrane surface receptors disrupts focal adhesion kinase (FAK) signaling pathways, thereby reducing substrate adhesion and modulating microenvironmental navigation.

### 4.2 Functional regulation of lactylation in hypoxic microenvironments

Hypoxia induces the Warburg effect, resulting in lactate accumulation that subsequently promotes histone lactylation through increased substrate availability. This metabolic‒epigenetic coupling regulates cellular proliferation via modulating the expression of proliferation‒associated genes and integration of key signaling pathways, including HIF‒1α and mechanistic target of rapamycin (mTOR) pathways. In various cell types, including glioma, non-small cell lung cancer (NSCLC), and esophageal cancer cells, lactylation has been shown to promote cell proliferation by regulating signaling axes such as HIF-1α and YTHDF2-BNIP3, implying a potentially similar role in MSCs (Dong et al., 2025; Chen et al., 2023; Zang et al., 2024; Yan et al., 2024).

Hypoxia‒induced lactylation differentially regulates MSCs proliferation in a time‒ and dose‒dependent manner. Acute hypoxia (≤48 h) enhances proliferation capacity via lactylation‒mediated Ki‒67 upregulation (Kastner et al., 2020). On the contrary, sustained hypoxia (>72 h) promotes ROS accumulation, leading to DNA damage and cell cycle arrest. This biphasic regulation is mirrored by lactate concentration effects: low‒dose lactate promotes mitotic activity, while high‒dose lactate induces gap 2/mitosis (G2/M) phase arrest via extracellular acidification. Therefore, elevating lactylation levels in MSCs via hypoxic preconditioning may represent a potential strategy to optimize their homing efficiency to injury sites.

Hypoxia‒induced lactylation coordinates MSCs migration through three complementary mechanisms (Selleri et al., 2016). First, lactylated transcription factors directly promote cell motility. As demonstrated by Yan et al. (2024), hypoxia‒mediated SRY-box transcription factor 9 (SOX9) lactylation enhanced stemness, migratory capacity, and invasiveness in NSCLC cells by activating EMT pathways (Yan et al., 2024). Secondly, lactylation dynamically modulates the SDF‒1/CXCR4 signaling axis, thereby amplifying chemotactic responses to injury‒associated chemokine gradients. Third, lactate increases matrix metalloproteinase (matrix metalloproteinase-2 [MMP‒2] and MMP‒9 activity), promoting extracellular matrix degradation and tissue barrier penetration (Wang CY. et al., 2022; Meng et al., 2024; Lin et al., 2016).

Hypoxic‒induced lactylation precisely modulates MSCs secretome via exosomal cargo modification and cytokine profile polarization. Specifically, lactylated proteins (including heat shock protein 90 [HSP90] and miR‒21‒5p) within exosomes significantly enhance their anti‒inflammatory and pro‒angiogenic capacities. Additionally, this metabolic‒epigenetic regulation promotes increased secretion of regenerative factors (including VEGF and interleukin-6 [IL‒6]) while suppressing pro‒inflammatory mediators expression at the transcriptional level (Selleri et al., 2016; Liu X. et al., 2024; Lopez et al., 2022).

Growing evidence demonstrates that lactylation exhibits distinct lineage‒specific regulatory effects in MSCs. Under hypoxic conditions, lactylation promotes osteogenic differentiation through wingless/integrated - beta-catenin (Wnt/β‒catenin) signaling potentiation and direct modification of osteogenesis‒related genes (Wu et al., 2023; Wu et al., 2024). Conversely, in adipogenic commitment, hyperglycemia‒induced lactate accumulation activates PPARγ via lactylation, driving lipid droplet formation and adipocyte differentiation (Kolodziej et al., 2019).

Futhermore, lactate metabolites function as key immunometabolic regulators that orchestrate immune cell polarization through distinct mechanisms: (1) promoting macrophage polarization via signal transducer and transcription 6/arginase 1 (STAT6/ARG1) pathway activation, and (2) suppressing T cell function via coordinated PD‒L1 upregulation and tryptophan depletion, collectively creating an immunosuppressive microenvironment (Pradenas et al., 2023; Chen et al., 2022).

## 5 Translation perspectives: targeting lactylation to enhance MSCs therapy

Accumulating evidence establishes lactylation as a pivotal regulator in disease pathogenesis, highlighting its dual potential as both a diagnostic biomarker and therapeutic target, particularly for cancer and metabolic disorders. Current therapy strategies focus on two primary approaches: (1) modulation of lactate metabolism through MCTs inhibition, and (2) direct targeting of histone lactylation. The MCT1 inhibitor AZD3965, currently in Phase I/II trials, demonstrates synergistic effects with immune checkpoint inhibitors (ICIs) by reducing TME lactate levels and potentiating anti‒tumor immunity (Beloueche-Babari et al., 2020). Similarly, MCT4 inhibition improves programmed cell death protein 1 (PD‒1) blockade efficacy in hepatocellular carcinoma (HCC) models, suggesting a potential therapeutic strategy for ICI‒resistant HCC patients (Chen et al., 2022). Studies have demonstrated that targeting lactylation-related pathways can effectively reverse therapeutic resistance in multiple malignancies, including colorectal cancer and bladder cancer (Li W. et al., 2024; Li et al., 2024d). Therefore, leveraging strategies from oncology that modulate lactate metabolism or directly inhibit lactylation could be applied in the MSCs field to enhance specific therapeutic functions.

Following strategies have revealed promising translational avenues for targeting lactylation in clinical applications, including personalized immunotherapy, chemoresistance reversal, anti‒angiogenic therapy, and cancer stem cell (CSC) targeting (He et al., 2024). By leveraging therapeutic strategies from the field of oncology, novel tools may be developed to optimize MSC-based therapies.

## 6 Discussion and future perspectives

### 6.1 Critial challenges and current research focus

Recent studies define a hypoxia‒MSC‒lactylation regulatory axis, revealing a coordinated metabolic‒epigenetic‒functional cascade that critically governs MSCs therapeutic efficacy. Several critical challenges remain unsolved in lactylation research. First, it is essential to acknowledge the current limitations in the MSCs field, particularly the scarcity of studies directly investigating lactylation in MSCs themselves. Many current mechanistic insights are extrapolated from cancer or immune cell models. Second, the technological challenges in detecting lactylation, such as the lack of highly specific, site-specific anti-lactylation antibodies, it hampers the precise mapping and validation of lactylation events. Third, the potential crosstalk among lactylation, acetylation, and methylation in disease progression demands systematic investigation. Finally, it remains exceptionally difficult to distinguish whether observed lactylation modifications are drivers of functional changes or merely correlative epiphenomena, necessitating the development of more sophisticated genetic and pharmacological tools for causal inference. Addressing these gaps will help elucidate lactylation’s role in pathogenesis and facilitate therapeutic development. Current research efforts primarily focused on two key areas: (1) mechanistic characterization through comprehensive lactylation site mapping via integrated metabolomic and epigenomic analyses, coupled with functional validation using gene editing or targeted pharmacological inhibition; and (2) preclinical development, involving systematic assessment of lactylation‒modulated MSCs functional modulation in established murine disease models.

Future research should focus on three key priorities to advance MSC‒based therapies: (1) network elucidation‒comprehensive mapping of tissue‒specific lactylation interactomes in MSCs to identify origin‒dependent regulatory networks; (2) clinical‒translation‒establishment of standardized lactylation levels as a critical quality attribute (CQA) during MSCs production to ensure batch consistency; and (3) combinatorial approaches‒strategic integration of lactylation modulation with biomaterial scaffolds or cytokine priming to synergistically enhance therapeutic efficacy.

### 6.2 Future perspectives: bridging discovery to therapy

Research on lactylation has reached a critical preclinical‒to‒clinical transition phase, with four transformative research directions emerging: (1) Mechanistic elucidation: Employing single‒cell multi‒omics to delineate spatio-temporal lactylation dynamics accross MSCs subpopulations and conducting clustered regularly interspaced short palindromic repeats (CRISPR)‒based functional genomics screens to identify lactylation‒modifying enzymes (“writers” and “erasers”); (2) Technological innovation: Developing lactylation‒specific fluorescent biosensors for real‒time visualization in living MSCs and creating artificial intelligence (AI)‒powered predictive models of lactylation‒mediated gene regulatory networks; (3) Clinical standardization: establishing quantitative lactylation thresholds as critical release criteria for MSC‒based products and implementing longitudinal lactylation biomarker tracking in clinical trial protocols; and (4) Epigenetic crosstalk: systematically investigating the interplay between lactylation, acetylation, and methionine metabolism in MSCs fate determination and engineering next‒generation “smart MSCs” with lactylation‒responsive genetic circuits for microenvironment‒adaptive tissue repair.

Outstanding Questions and Future Directions.

Technology and Specificity: How can we develop next-generation tools to overcome current detection limitations?

Causality and Correlation: What innovative experimental approaches can establish lactylation as a functional driver rather than a passive correlate in MSCs biology?

Epigenetic crosstalk: How can we deconvolute the interconnected regulation of lactylation and other PTMs?

Therapy window: How can we achieve cell-type or context-specific lactylation targets to ensure safety for MSC-based products?

## 7 Conclusion

Hypoxic preconditioning has emerged as an effective approach to enhance the therapeutic potential of MSCs, primarily through glycolytic reprogramming and subsequent lactate accumulation. Beyond its conventional role as a metabolic byproduct, lactate is now recognized as a key signaling molecule that regulates cellular functions via lactylation‒mediated PTMs‒a novel metabolic‒epigenetic regulatory axis. This hypoxia‒lactylation crosstalk critically regulates MSCs functionality through two key dimensions: mechanistic regulation and therapeutic application. At the mechanistic level, hypoxia‒lactylation crosstalk: (1) directs immunomodulatory polarization via lactylation‒dependent PD‒L1/IL‒10 upregulation, (2) enhances tissue repair capacity by activating pro‒angiogenic factors and extracellular matrix remodeling pathways, and (3) maintains stemness properties via SRY-box transcription factor 2/octamer-binding transcription factor 4 (SOX2/OCT4) lactylation‒mediated pluripotency network stabilization. Furthermore, the metabolic‒epigenetic synergy plays a pivotal role in modulating these mechanisms. Clinically, current therapeutic strategies targeting this axis encompass: (1) lactylation‒specific agents (e.g., small‒molecule inhibitors against LDH or MCTs to modulate lactylation dynamics), (2) combination therapies (e.g., integrating hypoxic preconditioning with biomaterial scaffolds), and (3) safety evaluation parameters (e.g., establishing lactylation thresholds as CQA).
